# Immunomodulatory treatment and surgical management of idiopathic uveitis and juvenile idiopathic arthritis-associated uveitis in children: a French survey practice

**DOI:** 10.1186/s12969-021-00626-x

**Published:** 2021-09-03

**Authors:** Julie Molimard, Christine Pajot, Priscille Olle, Alexandre Belot, Pierre Quartier, Florence Uettwiller, Chloé Couret, Valentine Coste, Camille Costet, Bahram Bodaghi, Pascal Dureau, Marion Bailhache, Pascal Pillet

**Affiliations:** 1 Pediatric diseases and Rheumatology, CHU Bordeaux, Bordeaux, France; 2 Department of Pediatric Nephrology, Internal Medicine and Hypertension, CHU Toulouse, Toulouse, France; 3 Department of Ophtalmology, Hôpital Purpan, CHU Toulouse, Toulouse, France; 4 Department of Pediatric Nephrology, Rheumatology, Dermatology, Reference centre for Rheumatic, AutoImmune and Systemic diseases in children (RAISE), Hôpital Femme Mère Enfant, CHU Lyon, Lyon, France; 5 Paediatric Hematology-Immunology and Rheumatology Department, Reference centre for Rheumatic, AutoImmune and Systemic diseases in children (RAISE), Hôpital Necker-Enfants Malades, APHP, Paris, France; 6 Department of Allergology and Clinical Immunology, Hôpital Clocheville, CHRU de Tours, Tours, France; 7 Department of Ophtalmology, Hôtel-Dieu, CHU Nantes, Nantes, France; 8 Department of Ophtalmology, CHU Bordeaux, Bordeaux, France; 9 Department of Ophtalmology, Hopital Pitié-Salpêtrière, APHP, Paris, France; 10 Pediatric Ophthalmology Department, Fondation Ophtalmologique Adolphe de Rothschild, Paris, France; 11 Department of Pediatric emergencies, CHU Bordeaux, Bordeaux, France

**Keywords:** Arthritis, Juvenile, Uveitis, Immunotherapy, Cataract, Glaucoma, Child

## Abstract

**Background:**

Surgeries for idiopathic uveitis and juvenile idiopathic arthritis-associated uveitis in children are complex because of the high risk of inflammatory postoperative complications. There is no consensus about treatment adaptation during the perioperative period. The objectives of this study are to report the therapeutic changes made in France and to determine whether maintaining or stopping immunosuppressive therapies is associated with an increased risk of surgical site infection or an increased risk of uveitis or arthritis flare-up.

**Methods:**

We conducted a retrospective cohort study between January 1, 2006 and December 31, 2018 in six large University Hospitals in France. Inclusion criteria were chronic idiopathic uveitis or chronic uveitis associated with juvenile idiopathic arthritis under immunosuppressive therapies at the time of the surgical procedure, operated before the age of 16. Data on perioperative treatments, inflammatory relapses and post-operative infections were collected.

**Results:**

A total of 76 surgeries (42% cataract surgeries, 30% glaucoma surgeries and 16% posterior capsule opacification surgeries) were performed on 37 children. Adaptation protocols were different in the six hospitals. Immunosuppressive therapies were discontinued in five cases (7%) before surgery. All the children in the discontinuation group had an inflammatory relapse within 3 months after surgery compared to only 25% in the other group. There were no postoperative infections.

**Conclusions:**

The results of this study show varying practices between centres. The benefit-risk balance seems to favour maintaining immunosuppressive therapies during surgery. Further studies are needed to determine the optimal perioperative treatments required to limit post-operative inflammatory relapses.

## Background

Idiopathic uveitis and juvenile idiopathic arthritis-associated uveitis are the first cause of uveitis in children [1–3]. The uveitis is anterior, bilateral, chronic, insidious, initially asymptomatic but it can be associated with poor visual prognosis without appropriate treatment [4, 5]. Juvenile idiopathic arthritis (JIA) is the most common rheumatic disease in children [6]. Its prevalence varies from 0.04 to 4/1000 in European countries [7]. JIA is associated with chronic uveitis in 10–40% of cases, especially in oligoarticular forms, in young girls with positive antinuclear antibodies (ANA) [4]. In some cases, uveitis may occur before arthritis [8]. Similar form exists without arthritis association called idiopathic uveitis [3]. Chronic intraocular inflammation is responsible for complications such as cataract, glaucoma, band keratopathy, posterior synechiae or macular oedema, many of these leading to loss of vision [1, 9]. Although the prognosis of this pediatric chronic non-infectious uveitis has considerably improved in recent years, due to earlier recognition, development of immunosuppressive agents and biologicals [10, 11], one third of these children will develop loss of visual acuity, and 5% of affected eyes will lose sight (visual acuity 20/200 or worse) [12]. Cataract and intraocular hypertension unresponsive to medical treatment frequently requires surgical management to prevent amblyopia and other sequelae. These surgeries are complex because of the high risk of postoperative complications because of underlying inflammation. Inflammation must be perfectly controlled to optimize surgical outcomes and avoid uveitis relapse [12, 13]. However, immunosuppressive therapies used during surgery may favour postoperative infection. There is no consensus about how to adapt treatment during the peri-operative period. Most authors recommend intensifying immunomodulatory treatments using topical or/and systemic corticosteroids [14–17]. In France, some centres stop biologic agents or methotrexate in order to prevent infections [18, 19]. The primary objective of this study is to describe the therapeutic adaptations used for ocular surgery of idiopathic uveitis and uveitis associated with JIA in children treated with immunosuppressive drugs in several French University Hospitals. The secondary objective is to determine whether maintaining or discontinuing immunosuppressive therapies is associated with an increase risk of surgical site infection or an increased risk of inflammatory relapse.

## Materials and methods

We conducted a retrospective cohort study between January 1, 2006 and December 31, 2018 in six large French University Hospitals (Bordeaux, Lyon, Nantes, Necker Enfants-Malades in Paris, Toulouse and Tours). Inclusion criteria were chronic uveitis associated with JIA or idiopathic uveitis, operated before the age of 16, treated by immunosuppressive therapies for at least 2 months before surgery and followed for at least 3 months after surgery.

The diagnosis of JIA had to be made by a paediatrician specialized in paediatric rheumatology and the diagnosis of uveitis associated with JIA or idiopathic uveitis by an ophthalmologist specialized in paediatric ophthalmology. Immunosuppressive therapies were defined by the code L04A of Anatomical Therapeutic Chemical classification. Surgeries were defined as an invasive procedure, excluding laser procedures.

Patients with acute uveitis and all uveitis with identified aetiology (infectious, traumatic, Behçet…) were excluded. Parents (or children) who expressed opposition to their child’s inclusion in the study were also excluded.

### Resources and data collection

Data from electronic medical records were used in all hospitals. We collected for each case: patient characteristics (sex, age), type of JIA, ANA presence, uveitis localisation and first manifestation of the disease (arthritis or uveitis). We also collected perioperative data (3 months before and 3 months after surgery) if available: rheumatologic examinations (presence of arthritis), ophthalmologic examinations (uveitis activity and complications) and treatments.

### Outcomes

Active uveitis was defined as the presence of cells ≥1+ in the anterior chamber. ANA were considered positive from 1/160. Inflammatory relapse was defined as the occurrence or the aggravation of uveitis or arthritis in the first 3 months after surgery. Postoperative infection was defined as the occurrence of an infection in the first 3 months after surgery.

### Statistical analyses

Descriptive statistics were reported using mean and standard deviation (SD) for continuous variables and using absolute frequencies and percentages for categorical variables. Comparative analyses were made using Student tests or Analysis of Variance tests for quantitative variables and Chi 2 tests or exact Fisher tests for qualitative variables. *p*-values of 0.05 or less were considered statistically significant. Statistical analyses were performed using SAS® University (SAS Institute, North Carolina, USA) and Excel® (Microsoft Corporation, Redmond, WA, USA).

### Ethical aspect

As an observational multicentric study using pre-existing data, patients and their parents were individually informed in writing to ensure their non opposition. Formal IRB was not required according to the legislation in our country.

## Results

A total of 76 surgeries (on 76 eyes) were performed on 37 children (27 girls and 10 boys). Some eyes were operated on several times. Mean age at surgery was 9.2 ± 3.3 years (range: 3.9–15.9). Sixty-seven surgeries (88%) were performed on children with anterior uveitis and nine (12%) on children with panuveitis. Fifty-two surgeries (68%) were performed on children with AJI-associated uveitis and 24 surgeries (32%) on children with idiopathic uveitis. ANA were positive for 70 surgeries (95%) and all children with idiopathic uveitis had positive ANA. There were 32 cataract surgeries (42%), 23 glaucoma surgeries (30%) and 12 posterior capsule opacification (PCO) surgeries (16%). Methotrexate associated with adalimumab (*n* = 42, 55%) was the most frequent immunosuppressive therapy used, followed by methotrexate alone (*n* = 13, 17%). Ophtalmologic remission was obtained before surgery in 65 out of 70 cases (93%). Surgery were performed after at least 3 months without intraocular inflammation (median 5 months) in 45 out of 64 surgeries (70%) of surgeries. Patient characteristics are shown in Table 1.

**Table 1:** Global patient characteristics and according to groups

Characteristics	Total *n* = 76		Discontinuation group *n* = 5		Maintenance group *n* = 71	
Age at diagnosis — yr (mean ±SD)	4.4 ±2.7		4.1 ±2.2		4.5±2.7	
Female — no. (%)	49	(64.5)	4	(80.0)	45	(63.4)
First manifestation — no. (%)						
Arthritis	38	(50.0)	2	(40.0)	36	(50.7)
Uveitis	31	(40.8)	2	(40.0)	29	(40.8)
Both	7	(9.2)	1	(20.0)	6	(8.5)
Uveitis — no. (%)						
JIA-associated uveitis	52	(68.4)	3	(60.0)	49	(69.0)
Idiopathic uveitis	24	(31.6)	2	(40.0)	22	(31.0)
ANA positive — no./total no. (%)^a^	70/74	(94.6)	5	(100)	65/69	(94.2)
Type of uveitis — no. (%)						
Anterior	67	(88.2)	4	(80.0)	63	(88.7)
Panuveitis	9	(11.8)	1	(20.0)	8	(11.3)
Age at surgery — yr (mean ±SD)	9.2 ±3.3		8.3±4.2		9.2±3.2	
Surgery — no. (%)						
Cataract	32	(42.1)	4	(80.0)	28	(39.4)
Posterior capsule opacification	12	(15.8)	1	(20.0)	11	(15.5)
Glaucoma	23	(30.3)	0	(0.0)	23	(32.4)
Needling	4	(5.3)	0	(0.0)	4	(5.6)
Material removal	3	(3.9)	0	(0.0)	3	(4.2)
Retinal detachment	2	(2.6)	0	(0.0)	2	(2.8)
Immunosuppressive therapies — no. (%)						
Methotrexate + Adalimumab	42	(55.3)	2	(40.0)	40	(56.3)
Methotrexate	13	(17.1)	0	(0.0)	13	(18.3)
Methotrexate + Infliximab	7	(9.2)	1	(20.0)	6	(8.5)
Adalimumab	4	(5.3)	1	(20.0)	3	(4.2)
Etanercept	4	(5.3)	0	(0.0)	4	(5.6)
Infliximab	2	(2.6)	0	(0.0)	2	(2.8)
Abatacept	1	(1.3)	1	(20.0)	0	(0.0)
Mycophénolic acid	1	(1.3)	0	(0.0)	1	(1.4)
Azathioprine + Adalimumab	1	(1.3)	0	(0.0)	1	(1.4)
Methotrexate + Abatacept	1	(1.3)	0	(0.0)	1	(1.4)
Corticoid eye drops — no./total no. (%)^a^	46/72	(63.9)	3/5	(60.0)	43/67	(64.2)
Oral corticoid — no. (%)	31	(40.8)	2	(40.0)	29	(40.8)
Ophthalmic remission — no./total no. (%)^a^	65/70	(92.9)	5/5	(100)	60/65	(92.3)
Preoperative adjustement— no./total no. (%)^a^						
Corticosteroids pulse	21/74	(28.4)	2/5	(40.0)	19/69	(27.5)
Increase oral corticosteroids	21/73	(28.8)	3/5	(60.0)	18/68	(26.5)
Peroperative adjustement — no./total no. (%)^a^						
Corticosteroids pulse	29/69	(42.0)	2/5	(40.0)	27/64	(42.2)
Subconjunctival injection of corticosteroids	39/62	(62.9)	4/5	(80.0)	35/57	(61.4)
Intracamerular corticosteroids	11/61	(18.0)	0/5	(0.0)	11/56	(19.6)
Intracamerular antibiotics	27/60	(45.0)	3/5	(60.0)	24/55	(43.6)
Antibiotics eyedrops	51/60	(85.0)	2/5	(40.0)	49/55	(89.1)
Local antineoplastic drugs	16/65	(24.6)	0/5	(0.0)	16/60	(26.7)
Postoperative adjustement — no./total no. (%)^a^						
Corticosteroids pulse	18/72	(25.0)	2/5	(40.0)	16/67	(23.9)
Increase oral corticosteroids	60/74	(81.1)	5/5	(100)	55/69	(79.7)
Corticosteroids eyedrops	61/62	(98.4)	4/5	(80.0)	57/57	(100)
Antibiotics eyedrops	59/59	(100)	5/5	(100)	54/54	(100)

^a^Data not available for some patients

Intravenous steroid pulses injections were used in 39 out of 71 surgeries (55%), oral steroids were introduced or increased in 62 out of 74 surgeries (84%). Patients received local steroids by intracameral injection in 11 out of 61 cases (18%), by subconjonctival injections in 39 out of 62 cases (63%) or ocular eye drops in 61 out of 62 cases (98%). Adaptations of perioperative treatments are detailed in Table 2. Steroid pulses therapy doses ranged from 2 mg/kg to 30 mg/kg (mean 12 mg/kg). Dosage of oral steroids during the perioperative period ranged from 0.3 up to 1.8 mg/kg/day. The steroids used for subconjonctival or intracameral injections were betamethasone, dexametasone or long-acting steroid (triamcinolone acetonide). One child received intravitreal dexamethasone implant. Immunosuppressive therapies were discontinued in five surgeries (7%), in two centres, in order to avoid infection. The interrupted treatments were abatacept, adalimumab, methotrexate (in association with infliximab which was not stopped) and adalimumab (2 patients; associated with methotrexate which was not stopped). The maximum period during which treatment was interrupted ranged from 5 weeks before surgery to 7 weeks after surgery.

**Table 2:** Perioperative treatment adjustment

Treatment	Surgeries *n* = 76
Preoperative — no./total no. (%)^a^	
Corticosteroids pulse	21/74 (28.4)
Discontinuation of immunosuppressive drugs	5/76 (6.6)
Peroperative — no./total no. (%)^a^	
Corticosteroids pulse	29/69 (42.0)
Subconjunctival injection of corticosteroids	39/62 (62.9)
Intracamerular corticosteroids	11/61 (18.0)
Intracamerular antibiotics	27/60 (45.0)
Antibiotics eyedrops	51/60 (85.0)
Local antineoplastic drugs	16/65 (24.6)
Postoperative — no./total no. (%)^a^	
Corticosteroids pulse	18/72 (25.0)
Increase oral corticosteroids	60/74 (81.1)
Corticosteroids eyedrops	61/62 (98.4)
Antibiotics eyedrops	59/59 (100)

^a^Data not available for some patients

Adaptation protocols were different in the six hospitals but also varied within some centres (Table 3). Uveitis relapse rate ranged from 14 to 67% depending on the hospital. Patient characteristics between patients with or without treatment discontinuation were similar (Table 1). The discontinuation group had only cataract surgeries (80%) and PCO surgeries (20%). Inflammatory relapse in the first 3 months after surgery occurred in 30% of surgeries. All children in the discontinuation group relapsed within 3 months of surgery (between one and 6 days after surgery), with three ocular relapses and two articular relapses, compared to only 25% in the other group with 18 ocular relapses and one articular relapse (between 1 and 76 days after surgery, median 7 days). Uveitis recurred in 60% of cases in the discontinuation group compared to only 25% in the maintenance group. There were no postoperative infections (Table 4).

**Table 3:** Adjustment of anti-inflammatory treatment by centre

	Center 1 *n* = 20	Center 2 *n* = 21	Center 3 *n* = 14	Center 4 *n* = 7	Center 5 *n* = 8	Center 6 *n* = 6	Total *n* = 76
Corticoids pulse — no./total no. (%)+a	5/20 (25.0)	9/21 (42.9)	9/14 (64.3)	4/5 (80.0)	6/7 (85.7)	6/6 (100)	39/71 (54.9)
Oral corticoids — no./total no. (%)^a^	15/20 (75.0)	18/20 (90.0)	12/14 (85.7)	3/6 (50.0)	8/8 (100)	6/6 (100)	62/74 (83.8)
Subconjonctival corticoids — no./total no. (%)^a^	10/19 (52.6)	5/13 (38.5)	9/14 (64.3)	3/3 (100)	6/7 (85.7)	6/6 (100)	39/62 (62.9)
Intracamerular corticoids — no./total no. (%)^a^	3/19 (15.8)	5/13 (38.5)	1/14 (7.1)	1/3 (33.3)	1/6 (16.7)	0/6 (0.0)	11/61 (18.0)
Corticoids eyedrops — no./total no. (%)^a^	19/20 (95.0)	10/10 (100)	14/14 (100)	6/6 (100)	6/6 (100)	6/6 (100)	61/62 (98.4)
Discontinuation of immunosuppressive drugs —no./total no. (%)	3/20 (15.0)	0/21 (0.0)	0/14 (0.0)	0/7 (0.0)	2/8 (25.0)	0/6 (0.0)	5/76 (6.6)

^a^Data not available for some patients

**Table 4:** Post-operative inflammatory relapses and infections

	Total *n*=76		Discontinuation group *n* = 5		Maintenance group *n* = 71	
	N	(%)	N	(%)	N	(%)
Relapse	23	(30.3)	5	(100)	18^a^	(25.4)
Uveitis relapse	21	(27.6)	3	(60.0)	18	(25.4)
Arthritis relapse	3	(3.9)	2	(40.0)	1	(1.4)
Infection	0	(0.0)	0	(0.0)	0	(0.0)

^a^A child has relapsed both at the joint and eye level

## Discussion

In this retrospective trial, practices were heterogeneous between centres and sometimes within the same centre, thus further illustrating the need for general recommendations. Maintenance of immunosuppressive therapy during surgery in patient with idiopathic uveitis and JIA-associated uveitis did not result in a significant number of infections and was associated with a lower rate of post-surgery uveitis flare.

Our study population was similar to the literature with a majority of girls presenting anterior uveitis, oligoarthritis and positive ANA [14–17]. Current practices are mostly focused on prevention of the inflammation. The literature suggests that preventing relapse is the cornerstone of uveitis prognosis [20]. Gregory et al. showed a time-dependant relationship between the presence of anterior chamber cells and the risk of visual loss [21]. For most studies, protocols required a period of at least 3 months with inactive intraocular inflammation before surgery [14, 15, 22], which is consistent with the data from our study. It is important to reinforce perioperative treatment in order to prevent post-operative inflammation as many recent guidelines suggest it, without standardized protocols. For glaucoma surgery, Wiese et al. proposed to increased topical dexamethasone eye drops 1 week before surgery and then continued after surgery, in association with topical prednisolone acetate eye drops [23]. For cataract surgery, Kulik et al. prescribed oral steroids for 2 days before surgery and intravenous hydrocortisone on the day of surgery. Subconjunctival injection of betamethasone was administered at the end of the surgical procedure [24]. Guindolet et al., administered intravenous steroids pulse therapy for two or 3 days before surgery, one pulse intraoperatively and three pulses during the three postoperative days [17]. In the French study conducted by Costet et al.*,* they proposed another therapeutic protocol: oral steroids initiated or intensified a few days before surgery (0.5 to 1 mg/kg/day), steroid pulse therapy of 10 mg/kg administrated intraoperatively with a subconjunctival and an intracameral injection of dexamethasone. Topical steroids were introduced postoperatively. Then, oral and local steroids were adapted to local inflammation [16]. In our study, the perioperative anti-inflammatory therapies were increased in all surgeries using intravascular, oral and/or local steroids. Children received at least one steroid pulse in almost half of the surgeries performed and oral intakes were increased in more than 80% of surgeries. Some children received subconjunctival injections of long-acting steroids or an intravitreal implant of dexamethasone. Their use in children is currently controversial, they are not evaluated as a perioperative management tool [25] and further studies are needed to quantify the risk of secondary glaucoma and steroid-induced cataract [26, 27].

In the literature, the risk of postoperative infection is often prevented by intracameral or subconjunctival antibiotics administered during surgery and antibiotic eye drops for the next few days [14, 15, 24]. In our study, patients had intracameral antibiotics in almost half of all the procedures, and antibiotic eye drops in all procedures.

A majority of authors do not stop immunosuppressive therapies before surgery and some even initiate immunosuppressive drugs before surgery to control inflammation [13, 28]. Holland et al. suggest that, unlike major surgery such as an orthopedic procedures, it is not necessary to stop methotrexate in children undergoing eye surgery [28]. Increased risk of infection during orthopaedic surgeries in adults on biologic agents is describe and this therapies are stopped in most cases [18, 19, 29]. However, this is not true for methotrexate, where several studies recommend continuing it during the perioperative period [29, 30]. In our study, only a few of pediatric rheumatologists stopped immunosuppressive therapies. However, there is no randomized data assessing the risk of infection after eye surgery performed under immunosuppressive therapies, probably due to the low prevalence of this kind of surgery. There were no infectious complications in our study. These results, regarding the safety of immunosuppressive therapies maintenance during surgery, are reassuring. Nevertheless, the incidence of endophthalmitis in cataract surgery being between 0.1 ‰ and 1 ‰, our study is not powered to allow us to identify a small increase in risk of endophthalmitis. The inflammatory relapse rate was high, especially in the discontinuation group in which all children relapsed after surgery, even though this group seems to have a stronger intensification of anti-inflammatory treatment. These results indicate the need to improve perioperative treatment adaptation in France. In this study, discontinuation of immunosuppressive therapies appears to be associated with an increased risk of relapse. The benefit-risk balance seems to be in favour of maintaining immunosuppressive therapies.

These results must be interpreted with caution given the small size of the cohort and the small number of surgeries in the discontinuation group, which does not allow us to perform statistical analysis. In addition, differences in surgical indications between the two groups could have influenced these results. This study was also limited by a significant amount of missing data, up to 20% for some variables. This is inevitable in retrospective studies, especially in patients followed by several specialists, with some data lost and some data not available from electronic medical records (non-computerised records or specific ophthalmologic data not available from electronic medical records). We chose to include idiopathic uveitis and JIA-associated uveitis as these chronic, anterior uveitis in children with positive ANA are very similar diseases in terms of complications, prognosis and treatment [2]. Many of these children develop joint damage after months or years [8, 31]. In addition, the frequency of children with idiopathic uveitis seems comparable in both groups. Our inclusion criteria included uveitis treated using immunosuppressive therapies for at least 2 months before the surgery and followed for at least 3 months after. This cut-off was chosen arbitrarily, considering that one to 3 months are needed for treatment to be effective and that discontinued treatments are generally resumed between one to 2 months after surgery. There are four patients who had two different surgeries within 3 months, which results in an overlap of relapse monitoring. We had decided not to exclude these patients with multiple surgeries. Only one of these patients relapsed during the overlap period, two and half months after the initial surgery. This results in an immortal time bias over a 15-day period.

To our knowledge, this is the first study that focuses on adaptation of immunosuppressive therapies during surgery for JIA-associated uveitis. The strength of this study is that it is a population-based cohort from six participating centres across the country, with the advantage of studying different practices and avoiding a single centre effect. Because of the rarity of this pathology, this is one of the largest series studying JIA-uveitis surgeries in children. In our days, few children undergo JIA-associated uveitis surgeries thanks to immunosuppressive therapies. By controlling inflammation, immunosuppressive drugs reduce the occurrence of complications and may even prevent the occurrence of uveitis [32]. Hence, the rate of blindness has decreased from 10 to 18% per affected eye, to less than 5% [5, 12, 33].

## Conclusions

Our study highlights the need of standardized ophthalmological protocols in France, resulting in varying surgerical outcomes and a high relapse rate. Nevertheless, our study is reassuring regarding the safety of immunosuppressive therapies maintenance during these surgeries. Further studies are needed with larger cohorts in order to precise postoperative relapse risk factors, optimal perioperative therapeutic adaptation and optimal surgical techniques.

## Data Availability

The datasets used and/or analysed during the current study are available from the corresponding author on reasonable request.

## Abbreviations

ANA: Antinuclear Antibodies
JIA: Juvenile Idiopathic Arthritis
PCO: Posterior Capsule Opacification
SD: Standard Deviation

## References

[1] Kump LI, Cervantes-Castañeda RA, Androudi SN, Foster CS (2005). Analysis of pediatric uveitis cases at a tertiary referral center. Ophthalmology..

[2] Angeles-Han ST, Lo MS, Henderson LA, Lerman MA, Abramson L, Cooper AM, et al. Childhood Arthritis and Rheumatology Research Alliance consensus treatment plans for juvenile idiopathic arthritis-associated and idiopathic chronic anterior uveitis. Arthritis Care Res. 2019;71(4):482–91. 10.1002/acr.23610. 10.1002/acr.23610 PMC6261704 29806733

[3] Holland GN, Denove CS, Yu F (2009). Chronic anterior uveitis in children: clinical characteristics and complications. Am J Ophthalmol.

[4] Bader-Meunier B, Filière de santé des Maladies Auto-Immunes et Auto- Inflammatoires Rares. Haute Autorité de Santé - Arthrites Juvéniles Idiopathiques [Internet]. [cited 2019 Jul 3]. Available from: https://www.has-sante.fr/portail/jcms/c_2801939/fr/arthrites-juveniles-idiopathiques

[5] Petty RE, Smith JR, Rosenbaum JT (2003). Arthritis and uveitis in children. Am J Ophthalmol.

[6] Gowdie PJ, Tse SML (2012). Juvenile idiopathic arthritis. Pediatr Clin N Am.

[7] Thierry S, Fautrel B, Lemelle I, Guillemin F (2014). Prevalence and incidence of juvenile idiopathic arthritis: a systematic review. Jt Bone Spine Rev Rhum.

[8] Heinz C, Mingels A, Goebel C, Fuchsluger T, Heiligenhaus A (2008). Chronic uveitis in children with and without juvenile idiopathic arthritis: differences in patient characteristics and clinical course. J Rheumatol.

[9] Carvounis PE, Herman DC, Cha S, Burke JP (2006). Incidence and outcomes of uveitis in juvenile rheumatoid arthritis, a synthesis of the literature. Graefes Arch Clin Exp Ophthalmol.

[10] Ramanan AV, Dick AD, Jones AP, McKay A, Williamson PR, Compeyrot-Lacassagne S (2017). Adalimumab plus methotrexate for uveitis in juvenile idiopathic arthritis. N Engl J Med.

[11] Quartier P, Baptiste A, Despert V, Allain-Launay E, Koné-Paut I, Belot A, Kodjikian L, Monnet D, Weber M, Elie C, Bodaghi B, ADJUVITE Study Group (2018). ADJUVITE: a double-blind, randomised, placebo-controlled trial of adalimumab in early onset, chronic, juvenile idiopathic arthritis-associated anterior uveitis. Ann Rheum Dis.

[12] Heiligenhaus A, Minden K, Föll D, Pleyer U (2015). Uveitis in juvenile idiopathic arthritis. Dtsch Ärztebl Int.

[13] Terrada C, Julian K, Cassoux N, Prieur A-M, Debre M, Quartier P, LeHoang P, Bodaghi B (2011). Cataract surgery with primary intraocular lens implantation in children with uveitis: long-term outcomes. J Cataract Refract Surg.

[14] Grajewski RS, Zurek-Imhoff B, Roesel M, Heinz C, Heiligenhaus A (2012). Favourable outcome after cataract surgery with IOL implantation in uveitis associated with juvenile idiopathic arthritis. Acta Ophthalmol.

[15] Nemet AY, Raz J, Sachs D, Friling R, Neuman R, Kramer M, Pandi SK, Sharma V, Assia EI (2007). Primary intraocular lens implantation in pediatric uveitis: a comparison of 2 populations. Arch Ophthalmol.

[16] Costet C, Andrèbe C, Paya C, Pillet P, Richer O, Rougier MB, Korobelnik JF, Coste V (2019). Chirurgie de la cataracte uvéitique non infectieuse de l’enfant : bilan des pratiques actuelles en France. J Fr Ophtalmol.

[17] Guindolet D, Dureau P, Terrada C, Edelson C, Barjol A, Caputo G, LeHoang P, Bodaghi B (2018). Cataract surgery with primary lens implantation in children with chronic uveitis. Ocul Immunol Inflamm.

[18] Goodman SM, Menon I, Christos PJ, Smethurst R, Bykerk VP (2016). Management of perioperative tumour necrosis factor α inhibitors in rheumatoid arthritis patients undergoing arthroplasty: a systematic review and meta-analysis. Rheumatol Oxf Engl.

[19] Ito H, Kojima M, Nishida K, Matsushita I, Kojima T, Nakayama T, Endo H, Hirata S, Kaneko Y, Kawahito Y, Kishimoto M, Seto Y, Kamatani N, Tsutani K, Igarashi A, Hasegawa M, Miyasaka N, Yamanaka H (2015). Postoperative complications in patients with rheumatoid arthritis using a biological agent – a systematic review and meta-analysis. Mod Rheumatol.

[20] Phatak S, Lowder C, Pavesio C (2016). Controversies in intraocular lens implantation in pediatric uveitis. J Ophthalmic Inflamm Infect.

[21] Gregory AC, Kempen JH, Daniel E, Kaçmaz RO, Foster CS, Jabs DA (2013). Risk factors for loss of visual acuity among patients with uveitis associated with juvenile idiopathic arthritis: the SITE study. Ophthalmology..

[22] Hawkins MJ, Dick AD, Lee RJW, Ramanan AV, Carreño E, Guly CM, Ross AH (2016). Managing juvenile idiopathic arthritis–associated uveitis. Surv Ophthalmol.

[23] Wiese K, Heiligenhaus A, Heinz C (2014). Trabekulektomie bei juveniler idiopathischer arthritis-assoziierter uveitis. Ophthalmol..

[24] Kulik U, Wiklund A, Kugelberg M, Lundvall A (2018). Long-term results after primary intraocular lens implantation in children with juvenile idiopathic arthritis-associated uveitis. Eur J Ophthalmol.

[25] Cordero-Coma M, Garzo I, Calleja S, Galán E, Franco M, Ruíz de Morales JG. Preoperative cataract surgery use of an intravitreal dexamethasone implant (Ozurdex) in a patient with juvenile idiopathic arthritis and chronic anterior uveitis. J AAPOS 2013;17(6):632–634, DOI: 10.1016/j.jaapos.2013.07.014. 10.1016/j.jaapos.2013.07.014 24215803

[26] Samra KA, Maghsoudlou A, Roohipoor R, Valdes-Navarro M, Lee S, Foster CS (2016). Current treatment modalities of JIA-associated uveitis and its complications: literature review. Ocul Immunol Inflamm.

[27] Asproudis I, Katsanos A, Kozeis N, Tantou A, Konstas AG (2017). Update on the treatment of uveitis in patients with juvenile idiopathic arthritis: a review. Adv Ther.

[28] Holland GN, Stiehm ER (2003). Special considerations in the evaluation and management of uveitis in children. Am J Ophthalmol.

[29] Goodman SM, Springer B, Guyatt G, Abdel MP, Dasa V, George M, Gewurz-Singer O, Giles JT, Johnson B, Lee S, Mandl LA, Mont MA, Sculco P, Sporer S, Stryker L, Turgunbaev M, Brause B, Chen AF, Gililland J, Goodman M, Hurley-Rosenblatt A, Kirou K, Losina E, MacKenzie R, Michaud K, Mikuls T, Russell L, Sah A, Miller AS, Singh JA, Yates A (2017). 2017 American College of Rheumatology/American association of hip and knee surgeons guideline for the perioperative Management of Antirheumatic Medication in patients with rheumatic diseases undergoing elective Total hip or Total knee arthroplasty. Arthritis Care Res.

[30] Grennan DM, Gray J, Loudon J, Fear S (2001). Methotrexate and early postoperative complications in patients with rheumatoid arthritis undergoing elective orthopaedic surgery. Ann Rheum Dis.

[31] Tugal-Tutkun I, Quartier P, Bodaghi B (2014). Disease of the year: juvenile idiopathic arthritis-associated uveitis—classification and diagnostic approach. Ocul Immunol Inflamm.

[32] Papadopoulou C, Kostik M, Böhm M, Nieto-Gonzalez JC, Gonzalez-Fernandez MI, Pistorio A, Martini A, Ravelli A (2013). Methotrexate therapy may prevent the onset of uveitis in juvenile idiopathic arthritis. J Pediatr.

[33] Bolt IB, Cannizzaro E, Seger R, Saurenmann RK (2008). Risk factors and longterm outcome of juvenile idiopathic arthritis-associated uveitis in Switzerland. J Rheumatol.

